# Unmasking residual cardiovascular risk: the paradoxical interaction between remnant cholesterol and calculated LDL-C in a tertiary-care cohort

**DOI:** 10.1186/s12944-026-02952-z

**Published:** 2026-05-02

**Authors:** Hazar Gözgöz, Seher Kabul, Özlem Gürsoy Doruk

**Affiliations:** 1 Department of Clinical Biochemistry, Türkoğlu Dr. Kemal Beyazıt State Hospital, Kahramanmaraş, Turkey; 2 Present Address: Faculty of Medicine, Department of Clinical Biochemistry, Dokuz Eylul University, Izmir, Turkey; 3 Department of Clinical Biochemistry, Bitlis State Hospital, Bitlis, Turkey

**Keywords:** Remnant cholesterol, Prevalent ASCVD discrimination, Friedewald equation, Sampson-NIH equation, Equation-dependent bias

## Abstract

**Background:**

Residual atherosclerotic cardiovascular disease (ASCVD) risk often remains even after low-density lipoprotein cholesterol (LDL-C) levels have been brought down to target levels. Remnant cholesterol (RC) and inflammation have been increasingly linked to the residual risk. We aimed to investigating whether the discriminative value of RC the ability of RC to discriminate and its claimed interactions with LDL-C are due to a real clinical phenotype or are affected by formula-dependent biases between the Friedewald and Sampson-NIH equations.

**Methods:**

We performed a cross-sectional analysis of consecutively tested adults (*n* = 3,342) using residual serum samples from routine clinical monitoring. To reduce analytical variability, all lipid profiles were analyzed using a single, dedicated reagent lot. We contrasted risk models with Friedewald-calculated versus Sampson-NIH-calculated LDL-C to assess equation-dependent differences. Lipid parameters, hemoglobin A1c (HbA1c), estimated glomerular filtration rate (eGFR), and C-reactive protein (CRP) were measured. ASCVD was defined using International Classification of Diseases, 10th Edition (ICD-10) codes. Missing covariate data were handled using multiple imputation by chained equations (m = 50), with additional complete-case sensitivity analyses for CRP-related models. To reduce bias, the observed ASCVD status was included as an auxiliary variable; the outcome itself was not imputed. The discriminative performance of nested logistic regression models was assessed through the pooled area under the receiver operating characteristic curve (AUC) and pooled DeLong p-values.

**Results:**

The primary clinical focus was the presence of documented pre-existing ASCVD diagnoses, identified in 11.4% of the cohort, while 9.4% of participants met the criteria for atherogenic dyslipidemia (AD). In the primary analysis with Friedewald LDL-C, we detected a statistically significant (*p* < 0.001) negative interaction between LDL-C and RC, while logCRP remained an independent correlate in the adjusted model. Interestingly, when we verified this using the more accurate Sampson-NIH equation to minimize the possibility that the result would be solely due to calculation bias, the paradoxical interaction was still statistically significant (*p* = 0.003) along with a strong model performance (AUC: 0.729). This indicates that the interaction is not entirely explained by the mathematical artifact of the Friedewald formula, but rather represents a consistent statistical pattern in this cohort.

**Conclusion:**

RC adds statistically significant value to risk discrimination. The continuous inverse relationship of LDL-C with high RC identifies a statistical pattern consistent with persistent atherogenic burden despite apparently optimal calculated LDL-C levels. Awareness of this potential suppressor effect may aid in refining risk stratification in tertiary-care settings.

**Supplementary Information:**

The online version contains supplementary material available at 10.1186/s12944-026-02952-z.

## Background

Despite targeted low-density lipoprotein cholesterol (LDL-C) reduction [1, 2], substantial atherosclerotic cardiovascular disease (ASCVD) risk remains [3]. This residual risk is increasingly attributed to triglyceride-rich lipoproteins and their remnants, measured as Remnant Cholesterol (RC) [4–8]. Such atherogenic burden frequently manifests as atherogenic dyslipidemia (AD), characterized by elevated triglycerides (TG) and low high-density lipoprotein cholesterol (HDL-C) [9]. Nevertheless, a methodological blind spot is usually overlooked in these high-risk phenotypes. In everyday clinical practice, LDL-C is mostly measured by the Friedewald formula which loses its accuracy when triglyceride levels increase [10]. This leads to a possible paradox in patients with high RC since their dyslipidemic profile makes the usual calculation of LDL-C unreliable. Therefore, some LDL-C and RC interaction patterns or discordances [11] seen may largely be equation dependent biases that have been brought about by the use of the formula. Hence, we thought that if we replace Friedewald with the more robust Sampson-NIH equation [10] these risk associations might change. Although this newer formula can give more accurate results in dyslipidemic samples, its effect on risk modeling based on RC is still not well understood. Inflammation, which can be assessed by biomarkers like C-reactive protein (CRP), is another validated dimension of residual risk [12].

## Methods

### Study design and ethics approval

This observational investigation examined a cohort of adults that underwent routine clinical biochemistry testing at Dokuz Eylül University Central Laboratory. Instead of performing a simple retrospective data extraction from a standard database, we established a tightly controlled laboratory-based workflow to limit pre-analytical and analytical confounding factors. During the entire study period, we measured consecutive lipid panels with a single, dedicated batch of Direct LDL-C reagent on the Beckman Coulter AU5800 platform.

This strict procedure enabled us to using a single reagent lot and a consistent analytical setup throughout the study period for the complete set of 3,342 subjects, thereby minimizing analytical drift and batch-to-batch variation that are frequently encountered in routine clinical data. The study used de-identified leftover samples according to the permission from Non-interventional Research Ethics Committee (Approval Number: 2025/35 − 18) that also waived the individual consent requirement.

### Patient population and enrollment

Those who participated in this study were recruited from a tertiary care center, representing a real-world cohort with prevalent metabolic comorbidities rather than an exclusively high-risk secondary prevention population.

Overall, 3,737 adult patients (age ≥ 18 years) were assessed for inclusion. Patients were enrolled if the physician in charge expected a laboratory workup that besides a full lipid profile also included hemoglobin A1c (HbA1c), CRP, and creatinine.

To direct the primary analysis to the aspect of chronic cardiometabolic risk, pre-specified exclusion criteria were introduced. Patients were removed from the study if they had experienced a cardiovascular event or major surgery within the last 3 months. In order to exclude acute infection or active systemic inflammation, people with CRP levels over 10 mg/L were also removed from the study. Furthermore, patients with end-stage liver disease, end-stage kidney disease (estimated glomerular filtration rate (eGFR) less than 15 mL/min/1.73 m²), and pregnancy were also excluded from the study.

**Fig. 1:**
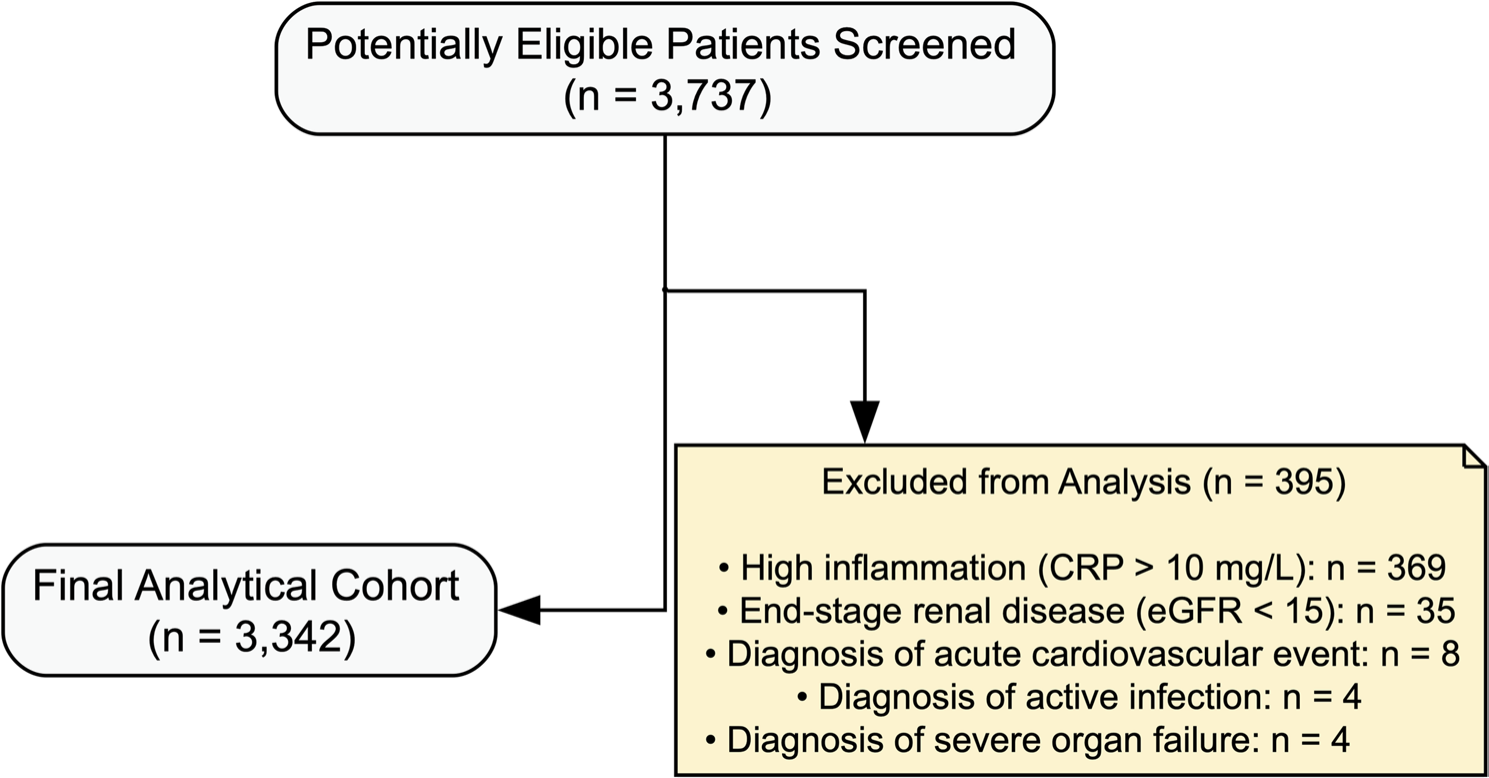
Study flowchart illustrating the patient selection process. The flowchart explains how the inclusion and pre-specified exclusion criteria were applied one after another to come up with the final analytical cohort of 3,342 patients. Note: eGFR limits for exclusion are expressed in mL/min/1.73 m²

The rationale for the exclusions is defined in Supplementary Tables 1, and after these 395 patients were removed, the remaining 3,342 patients formed the final analytical cohort. Figure 1 shows a flowchart of the patient selection process.

### Laboratory analyses

All laboratory measurements for the current study were carried out on serum samples at the Dokuz Eylül University Central Laboratory. Core biochemical tests were performed on an automated clinical chemistry analyzer (AU5800, Beckman Coulter, Inc., Brea, CA, USA). Lipid parameters were measured using enzymatic colorimetric assays. Specifically, direct LDL-C was determined via a homogeneous two-step enzymatic method. In the first step, a specific protecting agent shields LDL particles while non-LDL lipoproteins (HDL, VLDL, and chylomicrons) are enzymatically processed by cholesterol esterase and oxidase, with the resulting hydrogen peroxide eliminated by catalase. Following this, the protecting agent is released, catalase is inactivated, and the exposed LDL cholesterol reacts to form a photometrically measurable chromophore. According to the manufacturer’s data, these lipid assays exhibit excellent precision, with guaranteed repeatability coefficients of variation (CVs) of ≤ 3.0% (Beckman Coulter, catalog numbers: OSR6116 for TC, OSR60118 for TG, OSR6183 for direct LDL-C, and OSR6187 for HDL-C). Renal function (eGFR) was estimated using the Chronic Kidney Disease Epidemiology Collaboration (CKD-EPI) formula via an IDMS-traceable Jaffe reaction. CRP was measured immunoturbidimetrically, and HbA1c was quantified using an NGSP-certified HPLC cation-exchange method (Tosoh G11, Japan).

We estimated eGFR based on serum creatinine using the CKD-EPI formula. For the derived lipid metrics, Non-HDL-C was calculated as total cholesterol (TC) minus HDL-C. To isolate the effect of the LDL-C calculation method on model behavior, RC was defined across all models as TC − HDL-C − Direct LDL-C. To determine calculated LDL-C, we initially applied the standard Friedewald formula (TC- HDL-C -TG/5, all values expressed in mg/dL). To rule out method-induced artefacts in risk assessment, we also ran the Sampson-NIH equation. In a head-to-head comparison of these two equations within the hypertriglyceridemic subgroup (TG 150–400 mg/dL), we looked into the method-related disagreement. Remarkably, the Sampson-NIH formula yielded significantly higher estimates, producing a mean difference of as many as 4.9 mg/dL (Sampson-NIH minus Friedewald; *p* < 0.001) (Supplementary Table 2).

### Clinical diagnosis definitions

The primary clinical focus for this investigation was the presence of pre-existing ASCVD diagnoses. Patient diagnoses were extracted from the hospital’s electronic health records. To identify the disease, both structured diagnostic code fields and related free-text clinical notes were systematically examined for the International Classification of Diseases, 10th Edition (ICD-10) codes referring to the disease in question. ASCVD was considered present if the records contained any codes defining atherosclerotic disease already documented, e.g. I20-I25 (Ischemic heart diseases), I63-I69 (cerebrovascular diseases), G45 (transient cerebral ischemic attacks), or I70 (atherosclerosis/peripheral vascular disease).

### Definition of atherogenic dyslipidemia

AD phenotype was identified using the criteria of The National Cholesterol Education Program (NCEP) Adult Treatment Panel III (ATP III) [13], i.e., both elevated TG and low HDL-C occurring together: TG > 150 mg/dL and HDL-C < 40 mg/dL in males and < 50 mg/dL in females. The National Cholesterol Education Program Adult Treatment Panel (NCEP ATP III) criteria were selected because they represent a widely used clinical standard for metabolic dyslipidemia identification in daily practice.

### Statistical analyses

Python (version 3.10) with the pandas, numpy, scipy, statsmodels, and scikit-learn libraries was used to perform statistical analyses of the data. While the interaction model (Model 4 + INT) was the pre-specified primary analysisvariance inflation factors (VIFs), with VIF values <10 considered, other comparisons across subgroups were regarded as exploratory. Potential multicollinearity between variables in the final multivariable models was assessed using variance inflation factors (VIFs), with VIF values <10 considered acceptable. Regarding missing data, the primary multivariable analyses were performed on 50 multiply imputed datasets generated using multiple imputation by chained equations (MICE). Complete-case analyses involving CRP were additionally performed as conservative sensitivity analyses.

### Descriptive statistics and group comparisons

Characteristics at baseline of the studied population are expressed as mean ± standard deviation (SD) for continuous variables with normal distribution and as median [interquartile range (IQR)] for skewed variables. Categorical variables are expressed as counts and percentages (n, %), Wilson score method was used to calculate 95% confidence intervals (CIs).

Between groups with and without AD, group comparisons were done using the Mann-Whitney U test for continuous variables and Chi-square test for categorical variables. p-values less than 0.05 (two-sided) were considered statistically significant. The Benjamini-Hochberg false discovery rate (FDR) procedure was used to adjust p-values for multiple comparisons.

### Management of missing data

A marked difference was observed in the baseline characteristics of patients with and without CRP measurement confirming that data are not missing completely at random (Supplementary Table 3).

Using the scikit-learn IterativeImputer with RandomForestRegressor running maximum 20 iterations per dataset expected to be sufficiently converged (Supplementary Fig. 1).

To reduce bias and preserve predictor–outcome relationships, the observed ASCVD status was included as an auxiliary variable in the imputation model; the clinical outcome itself was not imputed. Prior to analysis, we evaluated the skewness of all continuous variables (Supplementary Table 4). Because routine CRP exhibited an extreme right-skewed distribution (skewness coefficient: 6.32), it was log-transformed to satisfy regression linearity assumptions. Interestingly, our methodological exclusion of cases with TG > 400 mg/dL effectively truncated the extreme right tail of the lipid distribution. Consequently, the skewness for triglycerides and derived remnant cholesterol remained at a mathematically acceptable level (1.24), and we carried out the models using standard scaling (Z-scores) for these variables without further log-transformation.The following regression modeling was run on each of the 50 imputed datasets. We combined area under the receiver operating characteristic curve (AUC) values using Rubin-based rules, and we pooled pairwise comparison p-values across imputations utilizing Fisher’s method.

### Method comparison (direct and calculated LDL-C)

To rule out calculation artifacts, we looked into the concordance between the direct and calculated LDL-C methods specifically in 3,252 complete pairs with TG levels ≤ 400 mg/dL. We strictly applied this cutoff because the conventional Friedewald formula becomes mathematically invalid and is automatically suppressed beyond this threshold, and including higher TG values would have artificially inflated the discordance rate with a known methodological artifact. Bland-Altman technique was employed for the visual representation while the main analysis of agreement depended on Deming regression (results are mentioned in the text further below) and Lin’s concordance correlation coefficient (CCC).

### Nested logistic regression models

In order to find out how much improvement the inclusion of each marker for prevalent ASCVD could bring to the model, five nested logistic regression models were built stepwise. Continuous variables were converted into standard scores (Z-scores). The comprehensive model (Model 4) explicitly incorporated baseline clinical covariates (age, sex, HbA1c, eGFR) alongside calculated LDL-C, RC, and logCRP. Finally, to evaluate the synergistic effect of these lipid parameters, we incorporated a statistical interaction term between calculated LDL-C and RC (denoted as ‘INT’) into this model to form our final analytical model (Model 4 + INT).

### Model performance and sensitivity analyses

The model performance was evaluated thoroughly in multiple aspects. Discrimination was assessed by the pooled Area Under the Receiver Operating Characteristic Curve (AUC). Calibration was assessed by the Brier score, calibration-in-the-large, calibration slope, and pooled calibration plots by 10 quantile bins. Decision Curve Analysis (DCA) [14] was applied to evaluate the potential clinical utility of the models. In this context, the ‘risk threshold’ refers to the minimum disease probability at which a clinician would consider intervention justified. Net benefit was calculated across a range of threshold probabilities (0.05 to 0.50) to determine whether model-based decision-making provided greater benefit than the treat-all or treat-none strategies; these net benefit curves were averaged across all imputations. The added value was measured with the help of the continuous Net Reclassification Index (cNRI) and Integrated Discrimination Improvement (IDI) [15]. Pre-planned sensitivity analyses included three sets of repeats of the entire modeling workflow: (1) in hypertriglyceridemic subjects (TG 150–400 mg/dL), (2) in non-negative RC patients (RC ≥ 0), and (3) the method-comparison where the Friedewald-LDL variable was swapped with Sampson-LDL. The third sensitivity analysis was particularly important to reveal whether the strong interaction terms in the main model were biological or merely equation dependent. The detailed metrics of these analyses are provided in the Results section and Supplementary Materials.

## Results

### Baseline characteristics of the study cohort

Filtering out ineligible cases left us with a final analytical cohort of 3,342 patients (Fig. 1). As detailed in (Table 1), the population was predominantly female and middle-aged. The patients exhibited a moderate cardiometabolic risk profile, as evidenced by a mean HbA1c level of 6.1 ± 1.3% (indicative of prevalent prediabetes/diabetes) and a median CRP of 2.2 [IQR 1.0-4.3] mg/L. While 9.4% of the participants were diagnosed with atherogenic dyslipidemia, the main clinical condition of interest, pre-existing ASCVD diagnoses, were identified in 11.4% (*n* = 380) of the cohort. In this cohort, as many as 23.7% (*n* = 788) of the patients presented with dysglycemia (HbA1c ≥ 6.5%). Other baseline variables, including routine lipid parameters and renal function, are comprehensively summarized in (Table 1) rather than repeated here. 

**Table 1:** Baseline demographic and clinical characteristics of the study cohort

Characteristic	Value (*n* = 3,342)	Missing *n* (%)
Demographics		
Age, years (mean ± SD)	56.7 ± 16.8	0 (0.0%)
Sex (Female), n (%)	1,941 (58.1%)	0 (0.0%)
Sex (Male), n (%)	1,401 (41.9%)	0 (0.0%)
Lipid Profile (Median [IQR])		
Total Cholesterol, mg/dL	203.0 [172.0–235.0]	2 (0.1%)
Triglycerides, mg/dL	122.0 [89.0–175.0]	3 (0.1%)
HDL-C, mg/dL	54.0 [46.0–64.0]	3 (0.1%)
Calculated LDL-C (Friedewald), mg/dL	119.6 [95.0–145.4]	90 (2.7%)
Calculated LDL-C (Sampson-NIH), mg/dL	122.4 [97.5–147.9]	3 (0.1%)
Direct LDL-C, mg/dL	133.0 [110.0–158.0]	0 (0.0%)
Non-HDL-C, mg/dL	148.0 [121.0–176.0]	3 (0.1%)
Remnant Cholesterol (RC), mg/dL	14.0 [9.0–19.0]	3 (0.1%)
Metabolic & Renal		
HbA1c, % and mmol/mol (mean ± SD)	6.1 ± 1.3 (43 ± 14)	19 (0.6%)
CRP, mg/L (median [IQR])	2.2 [1.0–4.3]	1,307 (39.1%)
eGFR, mL/min/1.73 m² (mean ± SD) Creatinine, mg/dL (median [IQR])	84.6 ± 24.7 0.84 [0.71–1.02]	4 (0.1%) —
Clinical Categories, n (%)		
Hypertension (Yes)	377 (11.3%)	—
Atherogenic Dyslipidemia (Present) Dysglycemia (HbA1c ≥ 6.5%)	314 (9.4%) 900 (24.3%)	—
CKD Stage		
G1	1,561 (46.7%)	—
G2	1,237 (37.0%)	—
G3a–G3b (Moderate)	465 (13.9%)	—
G4 (Severe)	75 (2.2%)	—

Data are presented as mean ± SD, median [IQR], or n (%).The 90 missing values for calculated LDL-C belong exclusively to patients with TG > 400 mg/dL, as the laboratory information system automatically suppresses the calculation beyond this threshold

Abbreviations:*SD *standard deviation, *IQR *interquartile range, *HDL-C* high-density lipoprotein cholesterol, *LDL-C* low-density lipoprotein cholesterol, *Non-HDL-C* non-high-density lipoprotein cholesterol, *HbA1c *glycated hemoglobin, *CRP *C-reactive protein, *eGFR *estimated glomerular filtration rate, *CKD *chronic kidney disease

### Methodological discordance in LDL-C measurement

**Fig. 2:**
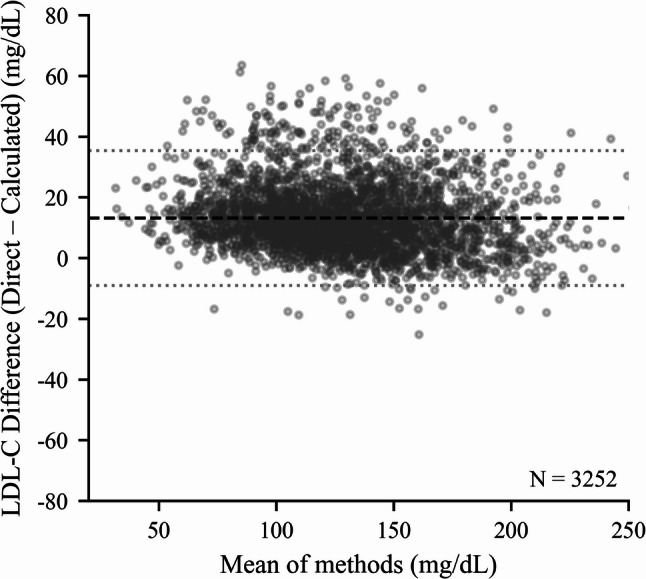
Bland-Altman plot quantifying the agreement between Direct LDL-C and standard calculated LDL-C. The findings indicate a systematic methodological disagreement thereby revealing the bias of standard formulas regarding high-risk cardiometabolic phenotypes

Before conducting the main analyses, the level of agreement between the unified Direct LDL-C and calculated LDL-C methods was examined in patients having TG levels < 400 mg/dL (*n* = 3,252 pairs). The remaining 90 individuals with TG > 400 mg/dL correspond exactly to the cases with missing calculated LDL-C values in Table 1, as the conventional Friedewald calculation is automatically suppressed by the laboratory information system (LIS) beyond this threshold.The Bland-Altman analysis (Fig. 2) disclosed a systematic bias in the conventional calculation methods. On average, Direct LDL-C exceeded Friedewald estimate by 13.2 mg/dL (95% Confidence Interval (CI): 12.8–13.5 mg/dL).

Significantly, upon isolating the specific hypertriglyceridemic subgroup (TG 150 to 400 mg/dL, *n* = 1,059), we found that the underestimation by the conventional formula was remarkably more pronounced. In this subset, the Friedewald calculation trailed the Sampson-NIH estimate by an average of 4.9 mg/dL (Supplementary Table 2).This reflects that in high-risk phenotypes, the standard Friedewald formula significantly underestimates the actual LDL-C level, thus, potentially generating artifacts in risk modeling. Thus, looking into the method agreement, Deming regression analysis (where X = Direct LDL-C and Y = Friedewald-calculated LDL-C) yielded the equation Y = 1.055X − 20.607**, and** this revealed the existence of both constant and proportional biases. Lin’s concordance correlation coefficient showed moderate to good agreement (CCC: 0.895), however, the methods lack parallelism as both the concordance and agreement are not perfect.

### Profile and correlates of atherogenic dyslipidemia

AD patients forming 9.4% of the cohort had significantly higher levels of biomarkers when compared to those without AD, even after correction for multiple testing (Table 2). The largest differences in the profile of AD were remarkably seen in TG (78.2% higher) and RC (38.4% higher). As detailed in Table 2, median RC, CRP, and HbA1c levels were all significantly elevated in the AD group compared to the non-AD group (*p* < 0.001 for all). Although the median RC in the AD group (18.0 mg/dL [IQR 13.2–23.8]) did not exceed the conventional 30 mg/dL high-risk threshold often cited for general non-fasting populations, this subgroup’s atherogenic risk is driven by a synergistically compromised phenotype. Specifically, the combination of elevated remnant lipoproteins, a markedly higher inflammatory burden (with 53.0% of AD patients presenting with high-risk CRP > 3.0 mg/L), and highly prevalent dysglycemia (42.4%) highlights the more adverse cardiometabolic profile of this specific AD subset. 

**Table 2:** Comparison of baseline characteristics according to atherogenic dyslipidemia (AD) status

Characteristic	AD Present	AD Absent	Adjusted *P*-value*
Lipid Profile (Median [IQR])	(*n* = 314)	(*n* = 3,028)	
Total Cholesterol, mg/dL Triglycerides, mg/dL	186.0 [160.0-210.0] 205.0 [174.0–276.2]	205.0 [174.0-238.0] 115.0 [85.0–157.0]	< 0.001 < 0.001
Calculated LDL-C (Friedewald), mg/dL	99.7 [77.6–121.4]	121.6 [97.2–147.8]	< 0.001
Calculated LDL-C (Sampson-NIH), mg/dL	102.3 [83.0–124.6]	124.4 [99.5–150.5]	< 0.001
Direct LDL-C, mg/dL	127.0 [106.2–145.0]	134.0 [110.0–160.0]	< 0.001
Remnant Cholesterol (RC), mg/dL HDL-C, mg/dL	18.0 [13.2–23.8] 39.0 [36.0–45.0]	13.0 [8.0–19.0] 56.0 [48.0–65.0]	< 0.001 < 0.001
Non-HDL-C, mg/dL	146.0 [123.0–168.8]	148.0 [121.0–178.0]	0.231
Cardiometabolic & Renal (Median [IQR])			
HbA1c, %, mmol/mol	6.2 [5.6–7.3] (44 [38–56])	5.7 [5.4–6.3] (39 [36–45])	< 0.001
CRP, mg/L	3.3 [1.9–5.6]	2.1 [1.0–4.1]	< 0.001
eGFR, mL/min/1.73 m²	87.0 [58.2–99.0]	88.0 [70.0–101.0]	0.080
Categorical Risk Factors, n (%)	(*n* = 314 / 284 / 166)	(*n* = 3,024 / 2,968 / 1,869)	Overall P-value**
Dysglycemia (HbA1c ≥ 6.5%)	156 (42.4%)	744 (22.3%)	< 0.001
Calculated LDL-C Strata (Friedwald)	(*n* = 284)	(*n* = 2,968)	< 0.001
< 70 mg/dL	51 (18.0%)	210 (7.1%)	
70–99 mg/dL	92 (32.4%)	616 (20.8%)	
≥ 100 mg/dL	141 (49.6%)	2,142 (72.2%)	
Chronic Kidney Disease Stage	(*n* = 314)	(*n* = 3,024)	< 0.001
G1 (eGFR ≥ 90)	146 (46.5%)	1,415 (46.8%)	
G2 (eGFR 60–89)	87 (27.7%)	1,150 (38.0%)	
G3a (eGFR 45–59)	38 (12.1%)	264 (8.7%)	
G3b (eGFR 30–44)	24 (7.6%)	139 (4.6%)	
G4 (eGFR 15–29)	19 (6.1%)	56 (1.9%)	
CRP Strata	(*n* = 166)	(*n* = 1,869)	< 0.001
< 1.0 mg/L (Low Risk)	23 (13.9%)	492 (26.3%)	
1.0–3.0 mg/L (Average Risk)	55 (33.1%)	705 (37.7%)	
> 3.1–10.0 mg/L (High Risk)	88 (53.0%)	672 (36.0%)	

Data are presented as median [IQR] or n (%). *Adjusted p-values for continuous variables were calculated using the Mann-Whitney U test followed by the Benjamini-Hochberg false discovery rate (FDR) procedure. **Overall p-values for categorical variables were calculated using the Chi-square test

*Abbreviations: AD *atherogenic dyslipidemia, *IQR *interquartile range, *LDL-C* low-density lipoprotein cholesterol, *HDL-C *high-density lipoprotein cholesterol, *HbA1c* glycated hemoglobin, *CRP* C-reactive protein, *eGFR *estimated glomerular filtration rate

Paradoxically, the higher risk group with AD had significantly lower median levels of calculated LDL-C (99.7 mg/dL vs. 121.6 mg/dL, *p* < 0.001). Similarly, direct LDL-C levels followed this trend, albeit with a less pronounced but still significant difference (127.0 mg/dL vs. 134.0 mg/dL, *p* < 0.001). Non-HDL-C (*p* = 0.231) and eGFR (*p* = 0.080) both showed no statistically significant difference between groups. There was a marked disparity in the prevalence of renal failure (CKD Stages G3-G4) between the AD (25.8%) and non-AD (15.1%, overall *p* < 0.001) groups. Similarly, in patients with CRP data, high-CRP AD patients significantly outnumbered high-CRP non-AD patients (53.0% vs. 36.0%, overall *p* < 0.001).

### Discordance between LDL-C and RC

Analyzing the prevalence of high RC (> 30 mg/dL) across LDL-C strata revealed substantial discordance. Using the Friedewald-based approach, nearly one-third of patients with optimal calculated LDL-C (< 70 mg/dL) exhibited high RC. Conversely, applying the methodologically corrected definition (TC - HDL-C - Direct LDL-C) eliminated this artificial discordance in our dataset, though this might be assay-dependent. However, true clinically relevant discordance remained at the < 100 mg/dL cutoff, where 10.4% of patients (*n* = 969) exhibited high RC (> 30 mg/dL).

**Fig. 3:**
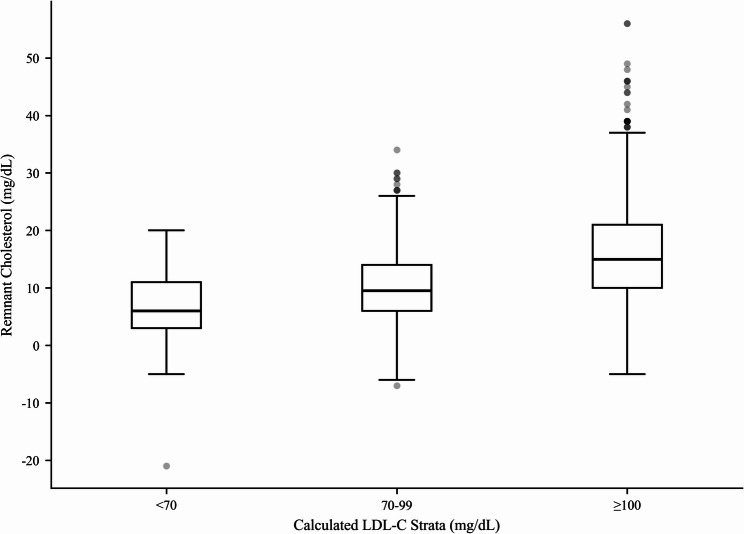
Box-and-whisker plots demonstrating the distribution of remnant cholesterol across different standard calculated LDL-C groups. The plot shows that many patients continue to have high atherogenic remnants that is a major cause of the residual risk even though calculated LDL-C levels appear to be controlled

Figure 3 illustrates this inconsistency, demonstrating that RC levels can be substantially elevated even in patients with optimal calculated LDL-C.

### Associative value of biomarkers for atherosclerotic cardiovascular disease

**Fig. 4:**
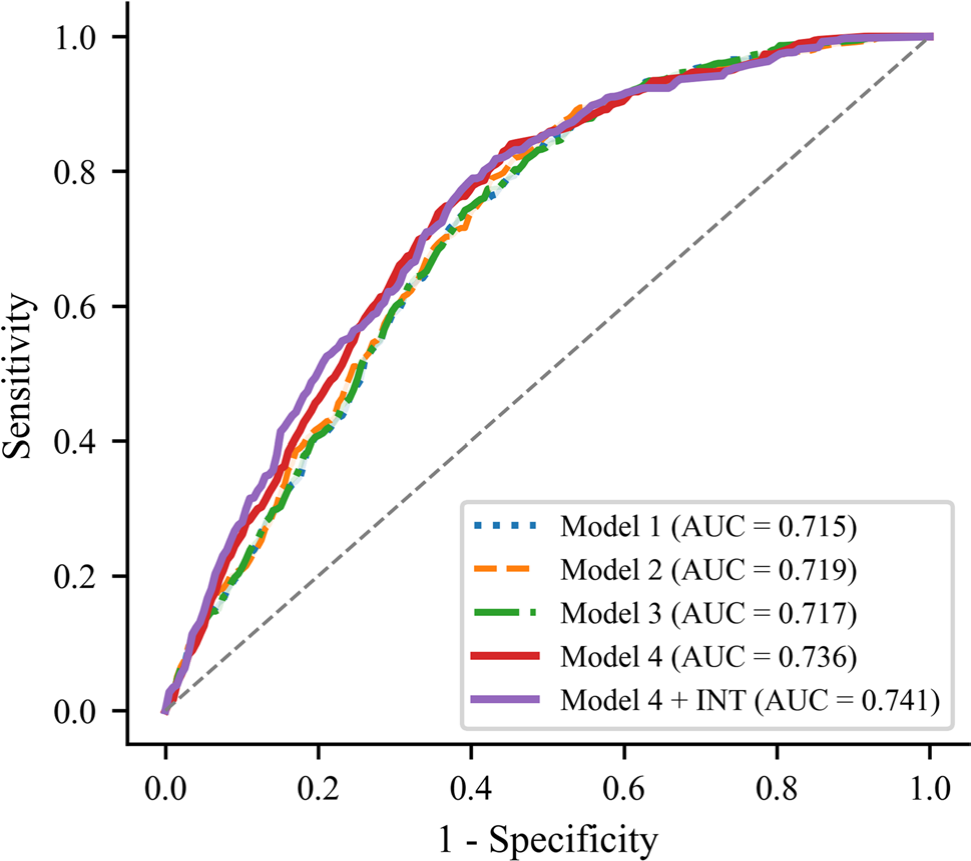
ROC curves for nested logistic regression models analyzing the presence of ASCVD. Model components: Model 1 (Age, Sex, HbA1c, eGFR); Model 2 (Model 1 + Calculated LDL-C); Model 3 (Model 1 + RC); Model 4 (Model 2 + RC + logCRP); Model 4 + INT (Model 4 + [LDL-C × RC interaction term]). The complete interaction model (Model 4 + INT) achieved the largest Area Under the Curve (AUC), thereby validating the additive level of discriminative power gained from the combination of RC and inflammatory markers

To figure out the associative value of these markers, we set up nested logistic regression models and pooled the results across 50 imputed datasets (Table 3). While the baseline clinical model (Model 1) achieved an AUC of 0.715, factoring in either standard calculated LDL-C (Model 2) or RC (Model 3) barely shifted the metric (AUCs 0.719 and 0.717, respectively). Remarkably, grouping inflammatory and lipid markers together in Model 4 drove the AUC up to 0.736, giving it a statistically significant edge over the standard LDL-C approach (*p* < 0.001). Building on this, the final interaction model (Model 4 + INT) yielded the highest discriminative power (AUC: 0.741); and although the absolute jump was modest, the interplay between LDL-C and RC proved highly significant (*p* < 0.001) (Fig. 4). 

**Table 3:** Discriminativeaccuracy of nested logistic regression models for prevalent ASCVD

Model	Model 1	Model 2	Model 3	Model 4	Model 4 + INT
AUC (95% CI)	0.715 (0.691–0.739)	0.719 (0.695–0.743)	0.717 (0.692–0.740)	0.736 (0.711–0.758)	0.741 (0.716–0.764)
Brier Score	0.0959	0.096	0.096	0.095	0.094
Hosmer-Lemeshow P-value	< 0.001	< 0.001	< 0.001	< 0.001	< 0.001
Sensitivity (%)	76.3	76.6	77.4	78.7	78.9
Specificity (%)	57.3	58.1	57.3	58.9	59.7
Accuracy (%)	59.5	60.2	59.6	61.2	61.9
PPV (%)	18.7	19.0	18.9	19.7	20.1
NPV (%)	95.0	95.1	95.2	95.6	95.7

Model components: Model 1 (Age, Sex, HbA1c, eGFR); Model 2 (Model 1 + Calculated LDL-C); Model 3 (Model 1 + RC); Model 4 (Model 2 + RC + logCRP); Model 4 + INT (Model 4 + [LDL-C × RC interaction term]). Diagnostic performance metrics (Sensitivity, Specificity, Accuracy, PPV, NPV) were evaluated at a clinically relevant risk threshold of 0.10, providing a standardized comparative baseline across all models

*Abbreviations: ASCVD *atherosclerotic cardiovascular disease, *AUC *area under the receiver operating characteristic curve, *CI *confidence interval, *INT *interaction term, *NPV *negative predictive value, *PPV *positive predictive value

### Independent correlates of atherosclerotic cardiovascular disease

To pin down the independent contribution of each biomarker, we looked into the pooled odds ratios (ORs) from our nested logistic models (Table 4). In the baseline model, advancing age, male sex, and paradoxically higher eGFR stood out as significant predictors of ASCVD. Pushing the analysis further into the comprehensive model (Model 4), calculated LDL-C showed a negative association with ASCVD, while logCRP emerged as a significant risk driver. Strikingly, rolling out the final interaction model (Model 4 + INT) exposed a robust and paradoxical interplay (*p* < 0.001). Within this fully adjusted framework, remnant cholesterol acted as a strong positive predictor, whereas Friedewald-calculated LDL-C maintained an inverse association. Pointing to a classic statistical suppressor effect, this pattern implies the model was mathematically correcting for the underestimated LDL-C values inherent in high-RC phenotypes. Finally, ruling out severe multicollinearity, all variance inflation factors (VIFs) remained comfortably below the conventional diagnostic thresholds (Supplementary Table 5). 

**Table 4:** Independent predictors of ASCVD (Pooled Odds Ratios)

Variable	Model 1	Model 2	Model 3	Model 4	Model 4 + INT
Age (per 1-SD)	2.34 [1.92–2.74]***	2.27 [1.90–2.71]***	2.32 [1.94–2.77]***	2.33 [1.95–2.79]***	2.40 [2.00–2.87]***
Sex (Male vs. Female)	2.54 [2.03–3.18]***	2.43 [1.94–3.06]***	2.53 [2.02–3.17]***	2.45 [1.95–3.09]***	2.32 [1.84–2.93]***
HbA1c (per 1-SD)	1.00 [0.89–1.11]	0.99 [0.89–1.11]	0.99 [0.89–1.11]	0.96 [0.86–1.08]	0.94 [0.83–1.05]
eGFR (per 1-SD)	1.25 [1.06–1.46]**	1.26 [1.07–1.48]**	1.25 [1.07–1.47]**	1.30 [1.11–1.53]**	1.33 [1.13–1.56]***
Calculated LDL-C (per 1-SD)	—	0.88 [0.78–0.99]*	—	0.84 [0.74–0.95]**	0.74 [0.64–0.84]***
Remnant Cholesterol (per 1-SD)	—	—	1.06 [0.98–1.14]	1.09 [0.98–1.21]	1.94 [1.49–2.53]***
logCRP (per 1-SD)	—	—	—	1.15 [1.02–1.29]*	1.10 [0.98–1.24]
Interaction (LDL×RC) (per 1-SD)	—	—	—	—	0.74 [0.65–0.85]***

All continuous variables were standardized (Z-scores) prior to analysis. In the interaction model (Model 4 + INT), the main effect odds ratios for calculated LDL-C and Remnant Cholesterol represent conditional effects (i.e., the estimated effect of one variable when the other is at its mean value), which explains the shift in magnitude from Model 4. *p < 0.05, **p < 0.01, ***p < 0.0

*Abbreviations: ASCVD *atherosclerotic cardiovascular disease, *HbA1c *glycated hemoglobin, *eGFR *estimated glomerular filtration rate, *LDL-C* low-density lipoprotein cholesterol, *CRP *C-reactive protein, *INT *interaction term

### Model performance: calibration and clinical utility

Besides discrimination (AUC), model calibration was examined for all imputed datasets (Supplementary Fig. 2). All models exhibited good calibration with pooled calibration slopes close to the ideal value of 1.0 (Model 4 + INT Slope: 1.00). The overall model fit, as indicated by the pooled Brier score, was roughly equal and slightly better for the most comprehensive model (Model 4 + INT Brier score: 0.094). Although the Hosmer-Lemeshow test yielded significant p-values (*p* < 0.001) for all models, this finding was interpreted cautiously given the known sensitivity of the test in large cohorts. Accordingly, calibration was assessed in conjunction with calibration plots, calibration slope, calibration intercept, and Brier score, which together suggested acceptable agreement between predicted and observed risks.

### Model performance: reclassification improvement

Stepping beyond basic discrimination, we quantified risk reclassification using cNRI and IDI metrics (Table 5). Mirroring the AUC trends, swapping LDL-C for RC alone (Model 3) failed to meaningfully improve classification over the standard baseline. However, wrapping RC, LDL-C, and logCRP together into Model 4 drove a highly significant and positive net reclassification for both cases and non-cases. More importantly, factoring in the interaction term (Model 4 + INT) triggered a substantial further leap in reclassification power compared to the main-effects model alone. By correctly reassigning risk across the cohort, this complete interaction framework delivered the highest overall improvement in both continuous net reclassification and integrated discrimination. 

**Table 5:** Reclassification improvement (NRI & IDI)

Comparison	cNRI	NRI (Events)	NRI (Non-Events)
Model 3 vs. Model 2	-0.7%	-7.9%	7.2%
Model 4 vs. Model 2	34.7%	18.9%	15.7%
Model 4 + INT vs. Model 4	25.8%	12.1%	13.7%

*Abbreviations: cNRI *continuous net reclassification index, *NRI *net reclassification index, *IDI *integrated discrimination improvement, *INT *interaction term. Model components: Model 1 (Age, Sex, HbA1c, eGFR), Model 2 (Model 1 + Calculated LDL-C), Model 3 (Model 1 + RC), Model 4 (Model 2 + RC + logCRP), Model 4 + INT (Model 4 + [LDL-C × RC interaction term])

### Decision curve analysis

**Fig. 5:**
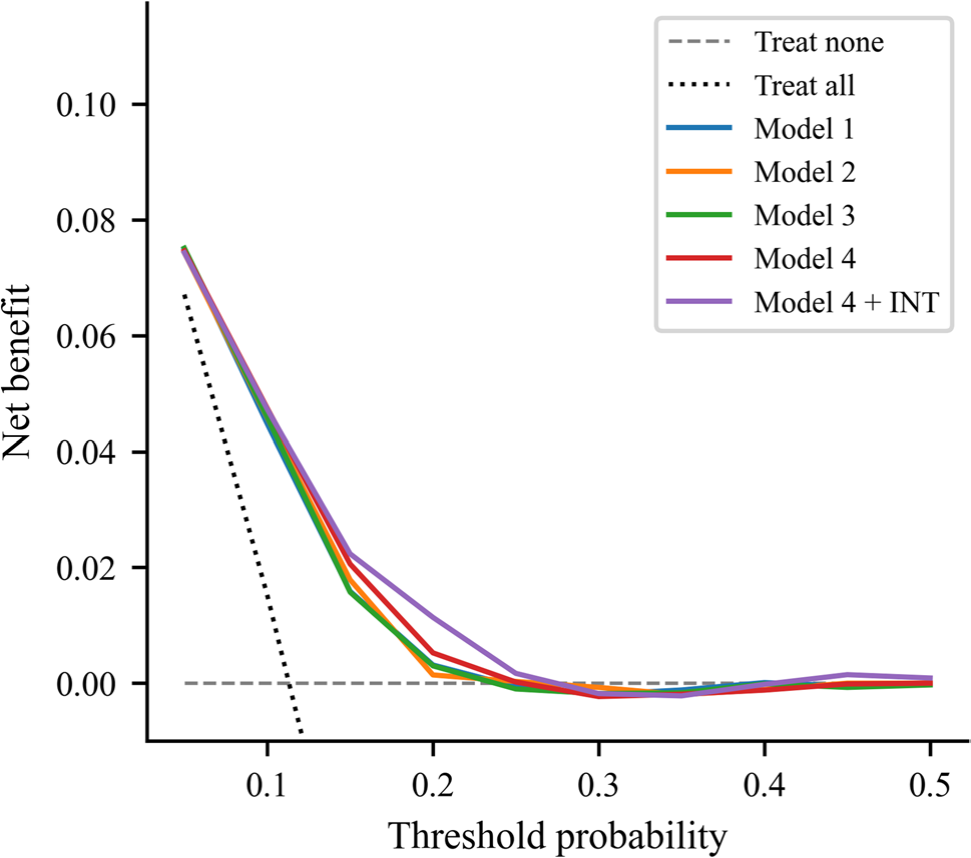
Decision Curve Analysis (DCA) benchmarking the clinical potential of the models. Model components: Model 1 (Age, Sex, HbA1c, eGFR); Model 2 (Model 1 + Calculated LDL-C); Model 3 (Model 1 + RC); Model 4 (Model 2 + RC + logCRP); Model 4 + INT (Model 4 + [LDL-C × RC interaction term]). The x-axis represents the risk threshold, defined as the minimum disease probability at which a clinician would decide to intervene. The horizontal grey dashed line represents the ‘treat-none’ strategy (assuming no patients have ASCVD), while the descending black dotted line represents the ‘treat-all’ strategy (intervening universally). A model is considered clinically useful if its curve lies above both these reference lines, indicating a higher net benefit. The plot demonstrates that the comprehensive interaction model (Model 4 + INT) provides consistently higher net benefits across most clinically relevant risk thresholds compared to the standard LDL-C-based model (Model 2)

We lastly evaluated the potential clinical usefulness of the models through DCA (Fig. 5). Throughout the entire range of reasonable risk thresholds (5% to 50%), the comprehensive models (Model 4 and Model 4 + INT) always delivered a higher net benefit than both the treat-all strategy and the standard model (Model 2). This suggests that utilizing the comprehensive models was connected with a higher net benefit (i.e., recognizing more true positives without raising false positives) than using the standard LDL-C model alone.

### Impact of the calculation method on risk estimates

We conducted rigorous sensitivity analyses to find out whether the observed interaction was a clinical reality or merely the result of equation-dependent bias. A complete-case analysis (*n* = 1,981) was performed on the subset of patients with no missing data for any of the Model 4 covariates. This reduction in sample size was primarily driven by the missing routine CRP values. This analysis seemed to indicate that the interaction could be weakened when using the Sampson-NIH equation (Supplementary Table 6). However, complete-case approaches tend to be biased due to selection in tertiary cohorts. Hence, to retain statistical power and cohort dynamics, the Sampson-NIH equation was applied throughout the fully imputed datasets (m = 50). Interestingly, in this primary robust model, the discriminative power increased (pooled AUC: 0.729), and the Sampson-LDL-C * RC interaction term was still very significant (*p* = 0.003) (Supplementary Table 6). This means that the interaction depends heavily on the cohort composition but remains strong when missing data are properly handled, thus indicating a real clinical phenomenon rather than just a Friedewald miscalculation.

We considered other confounding factors as well to strengthen our conclusions. A complete-case analysis without imputation supported the prediction power of RC (Supplementary Table 7). Also, an analysis of individuals with negative RC values showed that they had a specific phenotype of low TG and high HDL-C, although this agrees with known analytical limitations (Supplementary Table 8). Finally, the superior fit of the interaction model was supported by the lowest AIC and BIC values (Supplementary Table 9).

## Discussion

Our chief finding is that relying solely on calculated LDL-C leaves a substantial atherogenic burden undetected. By integrating RC and logCRP, we significantly improved ASCVD discrimination (*p* < 0.001), driving a cNRI of 25.8% and enhancing clinical utility in decision curve analysis. This predictive edge largely stems from utilizing Direct LDL-C rather than calculated LDL-C to determine RC. While European guidelines (EFLM/EAS) recommend directly measured LDL-C, routine practice often relies on Friedewald-calculated LDL-C due to economic constraints. Our findings support the practical relevance of the EFLM/EAS recommendations, illustrating how deviating from them generates systematic underestimations and suppressor effects in high-risk phenotypes. Furthermore, as visually indicated by the Receiver operating characteristic (ROC) curves (Fig. 4), the statistical discriminative edge of the interaction model appears particularly pronounced at lower sensitivity ranges. We fully acknowledge that in real-world ASCVD risk prediction, missing a high-risk patient carries a heavier penalty than over-flagging; therefore, maximizing sensitivity must be prioritized. Importantly, our Decision Curve Analysis (Fig. 5) resolves this exact clinical dilemma. It confirms that even at lower, conservative risk thresholds—which inherently favor high sensitivity to safely capture all potential patients at risk—the comprehensive model successfully maintains a consistent net benefit over standard approaches. Consequently, our results testify to the postulation that the combination of residual inflammatory risk (CRP) and RC risk provides a more comprehensive cardiovascular risk profile than LDL-C alone. Our data provide a clear demonstration of how equation-dependent bias distorts discordance analysis. When employing the Friedewald-based RC determination, 30.9% of patients with optimal LDL-C (< 70 mg/dL) appeared to have discordantly high RC. However, applying the methodologically corrected definition (using Direct LDL-C) completely eliminated this artificial discordance in our dataset. Consequently, high discordance rates reported in some prior studies might partly stem from statistical artifacts rather than novel biological phenotypes. A clinically meaningful, albeit smaller, true discordance remained only in the LDL-C < 100 mg/dL category (10.4% having RC > 30 mg/dL), confirming that LDL-C alone is inadequate for full risk profiling [16–18].

Our Bland-Altman analysis (Fig. 2) confirmed a systematic underestimation by calculated LDL-C, which reached clinically considerable levels in the AD phenotype (Table 2). Because the Friedewald formula’s assumptions are compromised in hypertriglyceridemic states [10], medical professionals may be erroneously reassured by falsely low calculated LDL-C values, thereby underestimating the actual atherogenic load in AD patients.

Besides lipids, inflammatory processes were also identified as a key player in residual risk. We found that AD patients had a lipoprotein pattern more atherogenic as well (a finding consistent with the role of AD in residual risk but also, among those for whom inflammatory biomarkers were available, a considerably greater inflammatory burden (53% vs. 36.0% with CRP > 3.0 mg/L). This is in line with clinical trial findings that suggest lipid and inflammation pathways being two correlated axes of risk [19–21].

Assessing the interaction between LDL-C and RC revealed a robust statistical suppressor effect. In the fully adjusted model, RC emerged as a strong positive predictor of ASCVD, whereas calculated LDL-C showed an inverse association. A sensitivity analysis utilizing the Sampson-NIH equation confirmed this interaction persisted (p = 0.003), indicating a genuine phenomenon rather than a miscalculation artifact. This inverse association likely reflects confounding by intensive lipid-lowering treatment typical in tertiary-care settings. Statins efficiently lower calculated LDL-C but have a limited effect on clearing triglyceride-rich remnants [22, 23]. Consequently, the negative coefficient for LDL-C in our model identifies a ‘treated high-risk phenotype’ rather than a biological protective effect, reiterating that LDL-C measurement alone is insufficient when RC levels remain elevated [16].

The unexpected positive association between eGFR and ASCVD risk initially appears paradoxical. In this relatively young and predominantly female cohort, the median baseline creatinine was closely clustered at 0.84 mg/dL. Consequently, this observation likely reflects the well-known mathematical inflation of estimated GFR in individuals with structurally lower muscle mass, rather than true hyperfiltration [24].

The downplaying of classic metabolic and inflammatory markers in our fully adjusted models was another highly informative finding. Even though logCRP was a significant predictor in Model 4, its strength of association dropped to a borderline level upon entering the LDL-C × RC interaction. Correspondingly, HbA1c did not emerge as a significant independent predictor. While a purely parsimonious statistical approach might suggest dropping these non-significant variables to simplify the model, we intentionally retained them. Our primary goal was not to create a minimalist prediction tool, but rather to observe how the independent predictive weights of established markers shift when RC and its interaction are introduced. The fact that logCRP and HbA1c lose their independent significance mathematically supports our hypothesis: RC likely captures the downstream atherogenic consequences of pathways shared with dysglycemia and inflammation. Retaining these variables ultimately proves that RC’s predictive power is an independent atherogenic vector, rather than just a proxy for missing HbA1c or CRP data. Recent studies support these findings, indicating that RC and inflammatory markers (like CRP) likely reflect intertwined atherogenic pathways [25–27]. Thus, our comprehensive models align with the notion that RC captures the downstream atherogenic consequences of the lipid component, encompassing risk pathways shared with inflammation.

Negative RC values, observed in ~ 2% of our cohort, consistently aligned with a specific low-TG/high-HDL-C phenotype. Rather than a calculation error, this artifact stems from the known measurement bias of homogeneous direct LDL-C assays under certain matrix conditions [28]. Importantly, excluding these technical aberrancies did not impact the overall prognostic performance (Supplementary Table 7).

### Limitations

Firstly, the cross-sectional nature of our study design does not allow us to establish causality. We can only show that certain factors are associated with prevalent ASCVD but not with incident cases. To determine whether these biomarkers can be used for predicting future risk, a longitudinal follow-up study would be necessary.

Secondly, a challenge in estimating RC using the formula ‘TC - HDL-C - Direct LDL-C’ is that it may turn out a negative number. In our study, we had negative RC values in a very small number of people (~ 2%, *n* = 71). Our analysis of this group showed that they were characterized by very low TG (mean 72.6 mg/dL) and very high HDL-C (mean 72.2 mg/dL). This is consistent with the known positive bias of homogeneous direct LDL-C assays in patients having high HDL/low TG profiles [28]. Furthermore, unmeasured Lipoprotein(a) [Lp(a)] likely contributed to this artifact, as Lp(a) is often inadvertently captured by direct LDL-C assays, artificially inflating the LDL-C value and driving the RC calculation into negative territory. Looking into our sensitivity analyses, however, excluding these technical outliers did not change the overall predictive power of the model. Thirdly, we based our lipid analysis on the conventional lipid panel. We did not determine Apolipoprotein B (ApoB), which is considered the most accurate measure of the total atherogenic particle burden, nor did we assess LDL particle number or size. The mismatch between RC and calculated LDL-C that we observed implies that ApoB would have clarified a lot. Nevertheless, although ApoB is a better marker of particle number, it is not always reimbursed or available in primary care settings. On the other hand, RC can be derived from the standard lipid panel at no extra cost, thus it is a very convenient means for widespread risk stratification. Moreover, while Direct LDL-C assays can be improved over the Friedewald formula in the case of hypertriglyceridemia, they are still prone to some bias coming from matrix effects and non-specific reactivity with other lipoprotein subfractions.

Fourthly, we could not include statin use and smoking status in our analysis because these data are rarely obtainable from LIS based extracts. Admittedly, statin therapy has an impact on lipid levels, but the true power of this ‘biochemistry-only’ approach is in its ability to perform opportunistic screening. According to our findings, it is possible that labs may raise the alarm about high-residual-risk phenotypes straight from the LIS database, even in the absence of medication history information. However, treatment and lifestyle factors not accounted for may have caused bias in the RC-ASCVD link. Most notably, aggressive lipid-lowering therapy in patients with established ASCVD may explain inverse associations for LDL-C and exaggerate the perceived relative importance of RC. Therefore, the effects reported should be seen as associations that can be distanced from control by indication.

Fifthly, our clinical diagnosis definition was based on ICD-10 codes which is a standard method used in large epidemiologic datasets; however, it remains subject to misclassification bias. Our data extraction was mainly LIS-based without full access to comprehensive clinical charts; therefore, there is a potential under-reporting of true ASCVD cases, and ICD codes may not always be the true reflection of the patient’s actual status.

Sixthly, our reliance on real-world LIS data resulted in a notable proportion (approximately 40%) of missing routine CRP values, as clinicians do not universally bundle this acute-phase inflammatory marker with standard outpatient lipid profiles. As demonstrated in our missingness analysis (Supplementary Table 3), these values were not missing completely at random, meaning that simply excluding these patients would have introduced severe selection bias. To overcome this, we employed Multiple Imputation by Chained Equations (MICE) utilizing a robust Random Forest algorithm (m = 50). While this state-of-the-art machine learning technique effectively handles complex non-linear missing data patterns and preserves cohort dynamics, the high proportion of imputed inflammatory data inherently remains a limitation that could influence the absolute precision of our comprehensive models. Finally, because this was a tertiary-care cohort enriched for metabolic disease, the generalizability of the findings may be limited. Although we rigorously carried out internal validation, we recognize the inherent instability of machine learning models depending on the specific subsample used for their development, which remains a major limitation.

## Conclusion

In this tertiary-care cohort, a comprehensive biochemical model incorporating RC and logCRP significantly improved ASCVD risk discrimination compared to standard LDL-C approaches. Our findings support the potential value of utilizing RC rather than relying solely on calculated LDL-C, suggesting that previously reported high discordance rates may be inflated by Friedewald-based calculation artifacts. In clinical chemistry, RC assessment is particularly relevant for patients with triglycerides between 150 and 400 mg/dL or those exhibiting discordant high RC (> 30 mg/dL) despite controlled LDL-C (< 100 mg/dL). However, the complex interactions driving residual risk require further longitudinal investigation to establish universal actionable thresholds. Despite logistical considerations related to cost and availability, our findings support the preference for directly measured LDL-C over calculated LDL-C for a more accurate estimationof remnant cholesterol.

## Supplementary Information

Supplementary Material 1.

## Data Availability

The datasets generated and/or analysed during the current study are not publicly available due to patient privacy and institutional ethics restrictions but are available from the corresponding author on reasonable request.

## Abbreviations

AD: Atherogenic dyslipidemia
ApoB: Apolipoprotein B
ASCVD: Atherosclerotic cardiovascular disease
AUC: Area under the receiver operating characteristic curve
CCC: Concordance correlation coefficient
CI: Confidence interval
CKD-EPI: Chronic Kidney Disease Epidemiology Collaboration
cNRI: Continuous net reclassification index
CRP: C-reactive protein
DCA: Decision curve analysis
eGFR: Estimated glomerular filtration rate
FDR: False discovery rate
HbA1c: Hemoglobin A1c
HDL-C: High-density lipoprotein cholesterol
ICD-10: International Classification of Diseases, 10th Edition
IDI: Integrated discrimination improvement
IQR: Interquartile range
LDL-C: Low-density lipoprotein cholesterol
LIS: Laboratory information system
NCEP ATP III: National Cholesterol Education Program Adult Treatment Panel III
OR: Odds ratio
RC: Remnant cholesterol
ROC: Receiver operating characteristic
SD: Standard deviation
TC: Total cholesterol
TG: Triglycerides
VIF: Variance inflation factor
