# Supplementary material for: Unmasking residual cardiovascular risk: the paradoxical interaction between remnant cholesterol and calculated LDL-C in a tertiary-care cohort
Source: Lipids Health Dis. 2026 May 2;25:155. doi: 10.1186/s12944-026-02952-z (PMC13281486; doi:10.1186/s12944-026-02952-z)
Supplement: Supplementary file 1 — Supplementary Material 1. [file 12944_2026_2952_MOESM1_ESM.docx]

#### **Supplementary Table 1.** Breakdown of exclusion criteria applied to the study population.

| **Exclusion Criterion** | **Number of Patients Excluded** |
| --- | --- |
| High inflammation (CRP > 10 mg/L) | 369 |
| End-stage renal disease (eGFR < 15 mL/min/1.73 m²) | 35 |
| Diagnosis of acute cardiovascular event | 8 |
| Diagnosis of active infection | 4 |
| Diagnosis of severe organ failure | 4 |
| Diagnosis of pregnancy | 0 |
| Total Exclusions | 395* |

**Note:** *Patients could meet more than one exclusion criterion simultaneously. **Abbreviations:** CRP, C-reactive protein; eGFR, estimated glomerular filtration rate.

**Supplementary Table 2.** Methodological discordance: Friedewald vs. Sampson-NIH Equation.

| **Method A** | **Method B** | **Mean Method A (mg/dL)** | **Mean Method B (mg/dL)** | **Mean Difference (A - B)** | **P-value** |
| --- | --- | --- | --- | --- | --- |
| Sampson-NIH | Friedewald | 131.4 | 126.5 | +4.9 | < 0.001 |
| Direct LDL-C | Friedewald | 150.1 | 126.5 | +23.6 | < 0.001 |
| Direct LDL-C | Sampson-NIH | 150.1 | 131.4 | +18.7 | < 0.001 |

**Note:** The analysis was carried out in a targeted subset of n=1,059 hypertriglyceridemic samples (TG 150–400 mg/dL). For this specific group, the mean direct LDL-C was 150.1 mg/dL, serving as the clinical benchmark for comparing the calculated estimates. A positive mean difference indicates that Method A yields significantly higher values than Method B. P-values were derived from paired t-tests.

**Supplementary Table 3.** Comparison of baseline characteristics between patients with missing vs. observed CRP data.

| **Characteristic** | **CRP Available (n=2,035)** | **CRP Missing (n=1,307)** | **P-value** |
| --- | --- | --- | --- |
| Age, years (Median [IQR]) | 58.0 [45.0–68.0] | 60.0 [49.0–70.0] | < 0.001 |
| eGFR, mL/min/1.73 m² (Median [IQR]) | 90.0 [76.0–102.0] | 84.0 [65.0–98.0] | < 0.001 |
| HbA1c, % (Median [IQR]) | 5.7 [5.3–6.3] | 5.8 [5.4–6.6] | < 0.001 |
| ASCVD Prevalence, n (%) | 45 (2.2%) | 335 (25.6%) | < 0.001 |
| Calculated LDL-C (Friedewald), mg/dL | 121.6 [89.0–141.0] | 117.0 [91.2–141.2] | < 0.001 |
| Remnant Cholesterol, mg/dL | 14.0 [9.0–20.0] | 13.0 [8.0–19.0] | 0.577 |
| Triglycerides, mg/dL | 121.0 [88.0–170.0] | 123.5 [90.0–179.0] | 0.095 |
| HDL-C, mg/dL | 55.0 [46.0–65.0] | 53.0 [45.0–61.0] | < 0.001 |

**Note:** P-values derived from Mann-Whitney U test (continuous) and Chi-square test (categorical). Calculated LDL-C values were derived using the standard Friedewald equation.

**Abbreviations:** IQR, interquartile range; CRP, C-reactive protein; eGFR, estimated glomerular filtration rate; HbA1c, hemoglobin A1c; ASCVD, atherosclerotic cardiovascular disease; LDL-C, low-density lipoprotein cholesterol; RC, remnant cholesterol.

**Supplementary Table** **4.** Skewness distribution of continuous clinical variables in the analytical cohort.

| **Clinical Variable** | **Skewness Coefficient** |
| --- | --- |
| **Routine CRP** | 6.32 |
| **HbA1c** | 2.29 |
| **Triglycerides** | 1.24 |
| **Remnant Cholesterol (Calculated)** | 1.24 |
| **Calculated LDL-C** | 1.04 |
| **Age** | -0.54 |
| **eGFR** | -0.76 |

**Note:** The analysis was carried out in n=3,252 paired samples following the exclusion of cases with triglycerides > 400 mg/dL. We worked out the skewness metrics using the unbiased Fisher-Pearson standardized moment coefficient. Significantly, values exceeding +3.0 are generally considered indicative of extreme right-skewness, which necessitates log-transformation for linear modeling. No data imputation was carried out, and models including CRP strictly utilized the complete-case subset (n = 2,035).

**Supplementary Table 5.** Assessment of Multicollinearity (VIF Diagnostics).

| **Predictor Variable** | **Variance Inflation Factor (VIF)** |
| --- | --- |
| Age | 2.13 |
| Sex (Male) | 1.06 |
| HbA1c | 1.12 |
| eGFR | 2.13 |
| Calculated LDL-C | 1.40 |
| Remnant Cholesterol | 6.21 |
| logCRP | 1.11 |
| LDL-C × RC (Interaction Term) | 5.45 |

**Note:** Interaction terms naturally exhibit higher VIFs, but values well below the conventional diagnostic threshold of 10 indicate that multicollinearity is not a significant concern.

**Supplementary** **Table 6**. Persistence of the LDL-C x RC Interaction across Datasets (Sampson-NIH Sensitivity Analysis).

| **Variable** | **Odds Ratio (95% CI)** | **P-Value** | **Interpretation** |
| --- | --- | --- | --- |
| Age | 2.42 [1.90-2.85] | < 0.001 | Significant |
| Sex (Male) | 2.35 [1.95-3.05] | < 0.001 | Significant |
| eGFR | 1.31 [1.05-1.50] | 0.010 | Significant |
| Sampson-NIH LDL-C | 0.75 [0.62-0.91] | 0.004 | Suppressor Effect |
| Remnant Cholesterol | 1.84 [1.32-2.58] | < 0.001 | Robustly Significant |
| Sampson-LDL x RC Interaction | 0.74 [0.61-0.90] | 0.003 | Interaction Persists |

**Note:** This multivariable logistic regression model (Model 4 + INT) utilizes the Sampson-NIH equation across all m=50 MICE datasets. The persistence of the inverse association for LDL-C and the highly significant interaction term (p=0.003) confirm that the suppressor effect is not a calculation artifact of the Friedewald formula. (In contrast, a complete-case analysis without imputation yielded an attenuated interaction [p=0.80], highlighting the susceptibility of complete-case approaches to selection bias in this tertiary cohort).

**Supplementary** **Table 7.** Sensitivity Analysis without Imputation Group.

| **Variable** | **OR (95% CI)** | **P-Value** | **Interpretation** |
| --- | --- | --- | --- |
| Remnant Cholesterol | 1.47 [1.14–1.88] | 0.002 | Robustly Significant |
| Calculated LDL-C | 0.78 [0.61–0.99] | 0.046 | Suppressor Effect Persists |
| Age | 3.48 [2.54–4.76] | < 0.001 | Significant |
| Sex (Male) | 1.89 [1.28–2.78] | 0.001 | Significant |
| eGFR | 1.39 [1.07–1.81] | 0.013 | Significant |

**Note:** Multivariable logistic regression model trained on complete-case data without MICE imputation.

**Supplementary Table 8.** Characteristics of Patients with Negative Remnant Cholesterol.

| **Parameter** | **Mean ± SD** | **Clinical Interpretation** |
| --- | --- | --- |
| Triglycerides | 72.6 ± 33.3 mg/dL | Very Low |
| HDL-C | 72.2 ± 15.2 mg/dL | High |
| Direct LDL-C | 103.5 ± 30.8 mg/dL | - |
| Remnant Cholesterol | -2.8 ± 2.7 mg/dL | Negative Artifact |

**Note:** While negative RC values were predominantly observed in patients with a low-TG / high-HDL phenotype, we cannot rule out the contribution of unmeasured Lipoprotein(a) [Lp(a)]. Since elevated Lp(a) is known to artificially inflate direct LDL-C measurements in homogeneous assays, its presence likely drove the calculated RC into negative values in this specific subset

**Supplementary Table 9.** Model Fit Statistics and Information Criteria.

| **Model** | **AIC** | **BIC** | **Pseudo R²** |
| --- | --- | --- | --- |
| Model 1 (Base) | 2183.1 | 2213.7 | 0.082 |
| Model 2 (+LDL) | 2180.1 | 2216.8 | 0.084 |
| Model 3 (+RC) | 2183.1 | 2219.8 | 0.083 |
| Model 4 (Comp) | 2174.6 | 2223.5 | 0.088 |
| Model 4 + INT | 2156.2 | 2211.2 | 0.097 |

**Note:** The significant drop in AIC for "Model 4 + INT" confirms improved model fit.

**
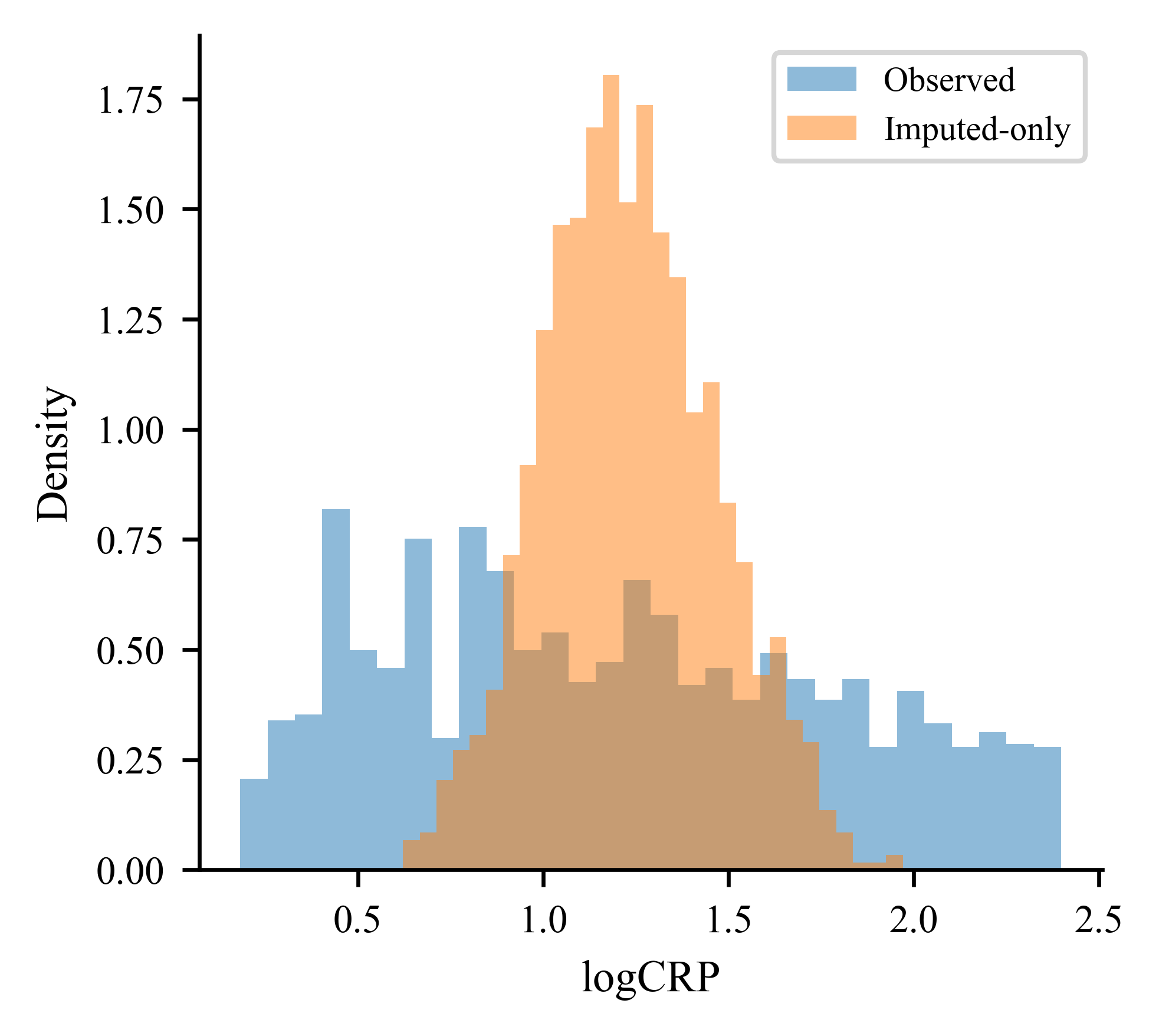
**

**Supplementary Figure 1.** Density distribution plot evaluating the multiple imputation model. The graph compares the distribution of observed (measured) logCRP values against the imputed logCRP values. The highly overlapping curves demonstrate that the imputation process accurately preserved the original variance and structural distribution of the dataset without introducing systemic bias.

**
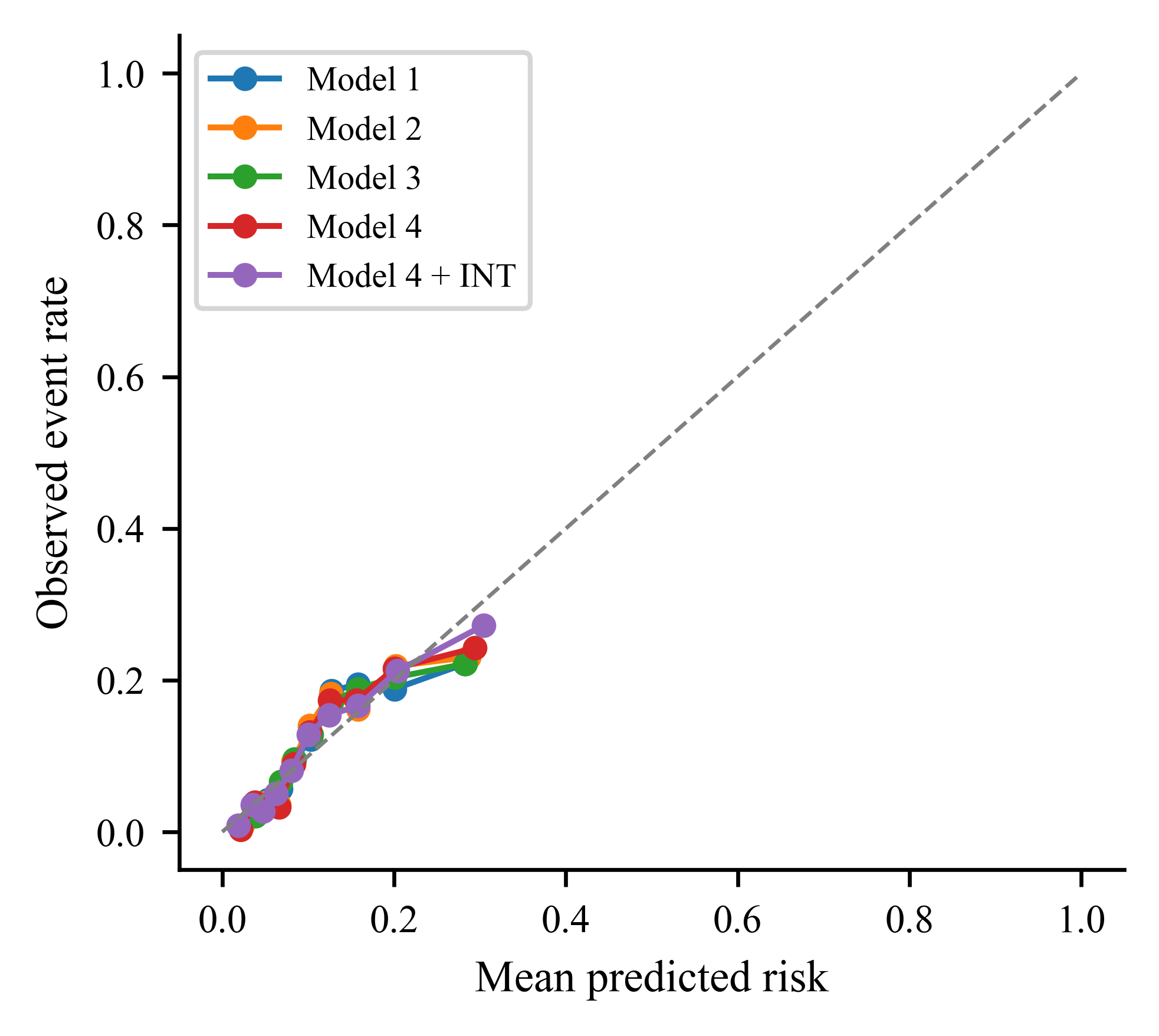
**

**Supplementary Figure 2. Calibration plots for the predictive models show how well the models performed on the imputed datasets.** The charts plot the predicted probabilities against the observed proportions of prevalent ASCVD and, as a result, all the models appear well-calibrated across the different quantiles.
